# Influence of Kersting’s (*Kerstingiella geocarpa*) groundnut proteins on the physicochemical, bioactive properties and storage stability of orange juice

**DOI:** 10.1016/j.heliyon.2021.e06246

**Published:** 2021-02-12

**Authors:** Omolayo R. Osungbade, Abiodun V. Ikujenlola, Saka O. Gbadamosi

**Affiliations:** Department of Food Science and Technology, Obafemi Awolowo University, Ile-Ife, Nigeria

**Keywords:** Ascorbic acid, Ferric ion-reducing, Metal chelating, Proteins isolate, Radical scavenging, Total antioxidant

## Abstract

Sweet orange ranks as one of the most acceptable fruit juices across the globe as it offers a healthy choice and pleasant taste to a wide spectrum of consumers. This makes it a promising vehicle for conveying functional ingredients into the human body. The present study was designed to produce functional orange juice by incorporating Kersting's groundnut proteins (isolate and hydrolysates) at different proportions (0.6, 0.8 and 1.0 w/v) into freshly produced sweet orange juice. The sample formulations were further analysed for changes in their physicochemical (pH, titratable acidity, total soluble sugars, ascorbic acid and total phenol content), sensory (appearance, colour, flavour, mouthfeel and overall acceptability), antioxidant (radical scavenging, metal chelating, ferric ion-reducing abilities, DPPH, total antioxidant) and antidiabetic (inhibition of α-amylase and α-glucosidase enzyme activities) properties as well as their storage stability over a 90-day storage period. The functional orange juice exhibited an improved physicochemical, antioxidant, antidiabetic and shelf life properties based on the respective protein concentrations used over the 90-day study period while the sample with the lowest proportion (0.6%) of functional ingredient had the highest sensory acceptability. Hence, the study concluded that Kersting's groundnut proteins could find useful applications in the formulation of functional orange juice.

## 1 Introduction

Recent improvements and modifications of plant proteins through isolation and hydrolysis have recorded remarkable accomplishments. This is not only as a cheap alternative to animal protein offering nutritional benefits, but also for the numerous health-promoting functions with guaranteed safety unlike those of synthetic origin (Aluko, 2008). Hence, modified proteins (protein hydrolysates) through enzymatic process could be used as natural sources of antioxidants in functional foods to maintain freshness, protect against oxidative damage and associated disease and extend shelf-life (Ajibola et al., 2011).

Functional foods are like traditional foods but possess established physiological benefits. However, their consumers’ reception is hinged largely on how and the form in which it is presented by the vehicle conveying them (Jonas and Beckmann, 1998; Poulsen, 1999; Siró et al., 2008**).** Although, functional foods range cut across different food products and classes including infant formula, beverages, confections, dairy, etc. (Ofori and Hsieh, 2013), however, offering the ease of storage and packaging appearance to meeting end-users needs, suitability with bioactive ingredients and other nutrients have placed beverage in the list of the most active groups (Sanguansri and Augustin, 2009; Wootton-Beard and Ryan, 2011; Kausar et al., 2012).

Orange juice is the liquid extracted from the endocarp of the sweet orange (*Citrus sinensis)* fruit (AIJN, 2012). It is high in certain functional components such as vitamin C, carotenoids and phenolic compounds (Martí et al., 2009). Galaverna and Dall'Asta (2014) reported that about a third of citrus fruit production goes for processing, the rest is eaten fresh with orange juice representing about 80% of the total citrus juice production.

Kersting's groundnut botanically referred to as *Macrotyloma geocarpum* [Harms] *Kerstingiella geocarpa* [Harms] (Maréchal and Baudet), Ground beans or Hausa groundnut is a subterranean legume belonging to the family *fabaceae* and with seeds resembling cowpea (Adu-Gyamfi et al., 2012). Nutritionally, *M. geocarpum* is utilized as edible seeds which are rich in protein (25%) and contain 42% of essential amino acids (mainly leucine, lysine, phenylalanine and valine) and 60–70% of carbohydrates with comparatively low level of antinutrients (Chikwendu, 2007). It is also a good source of mineral salts; calcium, magnesium, potassium, iron, etc. (Oyetayo and Ajayi, 2005). Other works have also been reported on its use as weaning formula (Aremu et al., 2011), fermentation (Abiola and Oyetayo, 2015), amino acid composition (Aremu et al., 2006), but not on its antioxidant and antidiabetic activity despite the reported chemical and nutritional composition.

In view of the health-promoting properties of functional foods in the management of oxidative-stress related disorders, most developed countries have consciously introduced functional beverages into their markets and this have gained consumers' acceptance. This study was therefore conducted to evaluate the effect of Kersting's groundnut proteins on the physicochemical, antioxidant, antidiabetic properties of orange juice with a view to producing a functional drink.

## 2 Materials and methods

### 2.1 Source of materials

Dried, fully matured Kersting's groundnut seeds were bought from a local market in Abakaliki, Ebonyi state, Nigeria where it was stored in woven sandbags (after about 6–8 months from harvest and drying). Fully-ripe orange (*Citrus sinensis cv. Valencia*) fruits were purchased within 4 h of harvest (for immediate use in the laboratory) from a local farm in Ile-Ife, south-western Nigeria. All chemicals and reagents used were of analytical grade and were purchased from Sigma and Aldrich Chemical company, USA.

### 2.2 Methods

#### 2.2.1 Preparation of defatted flour

Kersting's groundnut defatted flour was prepared from the whole full fat flour Kersting's groundnut using the method of Sathe (1994). The seeds were milled and sieved through 600 μm Laboratory test sieve and subsequently defatted with cold (4 °C) acetone (flour to solvent ratio 1:5 w/v) with constant magnetic stirring provided. The slurry was filtered off and the defatted flour was air-dried at room temperature and kept at 4 °C until used.

#### 2.2.2 Preparation of Kersting's groundnut protein isolate

Kersting's groundnut protein isolate was prepared from the defatted flour by a method described by Gbadamosi et al. (2012). A certain weight of the defatted flour was dispersed in an amount of distilled water to give 1: 10 flour to liquid ratio. The extraction proceeded with gentle stirring at 30 °C for 3 h while maintaining pH 10 (at which protein is most soluble). Non-solubilized materials were removed by centrifugation at 3500 × g for 10 min. The protein in the extract was precipitated by dropwise addition of 0.1 N HCl with constant stirring until the pH was adjusted to pH 4.0 (at which the protein is least soluble). The mixture was centrifuged at 3500 × g for 10 min and the precipitate washed twice with distilled water and the pH adjusted to 7.0 using 1 M NaOH, centrifuged and then freeze-dried and stored (as IS) at -4 °C for further use.

#### 2.2.3 Preparation of Kersting's groundnut protein hydrolysate

Kersting's groundnut protein hydrolysate was prepared by using pepsin, pancreatin and trypsin - proteolytic enzymes according to the method reported by Omoni and Aluko (2006). Fifty grams of dried Kersting's groundnut protein isolate was dispersed in 1000 ml of distilled water (5 % w/v). The pepsin-Kersting's groundnut protein hydrolysates (PsKPH) was prepared by adjusting the pH of the Kersting's groundnut protein isolate's slurry to 2.0 (using 0.1 N HCl) and the mixture was incubated at 37 °C followed by the addition of pepsin (1.8 g of pepsin powder; that is, 4 % w/w protein isolate (90.48 %). Similar method was followed for the pancreatin-Kersting's groundnut*-*protein hydrolysate (PcKPH) and trypsin-Kersting's groundnut*-*protein hydrolysate (TpKPH) but at pH of 7.5 and temperature of 40 °C and 50 °C respectively.

The digestion was carried out for 4 h keeping the temperature constant and pH maintained by adding 1 M NaOH or HCl when necessary. The reaction was terminated/stopped by adjusting the pH to 4.5 (with 1 M NaOH or HCl), then placing the mixture in boiling water for 20 min then placed on ice-bath for 1 h. The mixture was then centrifuged (7000 × g, 4 °C for 30 min and the resulting supernatant was freeze-dried to produce the respective enzyme-hydrolysed Kersting's groundnut protein hydrolysates (labelled as -PE, -PA and -TR respectively). These were stored at -4 °C until needed.

#### 2.2.4 Extraction of juice from orange fruit and fortification with kersting's groundnut proteins

The fruits were sorted, cleaned and washed under running water. The cleaned fruits were peeled to remove the pericarp and seeds with a sharp stainless-steel knife. Extraction of juice from orange fruit was carried out as described by Akusu et al. (2016). The fruit juice was extracted manually followed by filtering with a double layered filtering mesh to obtain clear juice. A portion of the juice was taken as control while the remaining was divided into 12 portions from which 3 portions each was fortified with different concentrations (0.6, 0.8 and 1.0 weight (g): volume (ml) based on the response from the preliminary sensory evaluation conducted) of Kersting's groundnut protein isolate and hydrolysate fractions. The fortified juice was packaged immediately in sterilized air-tight, plastic bottles and pasteurised at 80 °C for 10 min. This was cooled under running water and stored at ambient (32 °C) for a period of 3 months during which further analyses were conducted at intervals of 2 weeks.

#### 2.2.5 Physicochemical properties of functional orange juice

##### 2.2.5.1 pH

This was determined using a digital pH meter (pHs-2F, Harris, England) according to AOAC 981.12 (2000) method.

##### 2.2.5.2 Total soluble sugar (Brix)

This was determined using the hand-held sugar refractometer according to AOAC 932.12 (2000) method.

##### 2.2.5.3 Titratable acidity (TTA)

This was determined according to the AOAC 942.15 (2000) method.

Ten millilitres of the juice was pipetted into a conical flask and 25 ml of distilled water was added. Three drops of phenolphthalein were added (as an indicator) and the mixture was titrated against 0.1N KOH. Titration continued until a pink colouration was observed and the corresponding burette reading taken. Blank titration was carried out by replacing the sample with distilled water. TTA was calculated in Eq. (1) as:

(1) $$\%\;TTA=\;\frac{\left(molarlity\;of\;NaOH\;\times \left(Titre\;of\;sample-blank\right)\times 0.06404\right)}{mL\;of\;sample}\times 100\;\left(\%\right)$$

where 0.06404 = ml equivalent of citric acid.

##### 2.2.5.4 Ascorbic acid content

This was estimated by titrimetric method described by Rekha et al. (2012).

Exactly 5 ml of standard ascorbic acid (100 μg/ml) was measured into a conical flask containing 10 ml 4% oxalic acid. The mixture was titrated against the 0.0005M of 2, 6-dichlorophenol indophenols dye. Distilled water was added to the final volume of 1000 ml). The appearance and persistence of pink colour for 30 s was taken as the end point. The quantity of dye consumed (Va ml) was equivalent to the amount of ascorbic acid. Exactly 5 ml of sample (prepared by taking 5 ml of juice in 100 ml 4% oxalic acid) was measured inside a conical flask containing 10 ml 4% oxalic acid and titrated against the dye (Vb ml). The amount of ascorbic acid was calculated using the Eq. (2) as;

(2) $$Ascorbic\;acid\;\left(mg/ml\right)=\frac{x\;mg\;}{Va}\;\times \frac{V\;b\;}{15\;ml}\;\times \;\frac{100\;mL\;}{mL\;of\;sample\;used\;for\;analysis}$$

*x (mg)* = quantity of ascorbic acid dissolved in a known volume of oxalic acid.

*Va* = volume of dye consumed by the sample.

*Vb* = volume of dye consumed by the sample.

15 ml = total volume of sample and oxalic titrated.

100 ml = volume of oxalic acid solution used in dissolving the sample.

##### 2.2.5.5 Total phenol content

This was estimated following the procedure described by Singleton and Rossi (1965) and modified by Gulcin et al. (2003) using the Folin Ciocalteu's phenol reagent. A 10-fold dilution of Folin–Ciocalteu reagent was prepared just prior to use. To 100 μl of the juice was added 900 μl of distilled water to give 10-folds dilution. Two hundred microliters (200 μl) of freshly prepared diluted Folin-Ciocalteu's phenol reagent was added and the mixture was vortexed. After allowing the mixture to equilibrate for 5 min, the reaction was then neutralized with 1.0 ml of 7% (*w/v*) Na_2_CO_3_ solution. After 2 h of incubation at room temperature, the absorbance was measured at 750 nm. A standard curve was created with a linear range of 0.0–0.1 mg/ml using Gallic acid as the standard. The results were expressed as milligram Gallic acid equivalent (mgGAE/ml) of juice by extrapolation from the standard curve. Distilled water was used as blank.

#### 2.2.6 Antioxidant properties of functional orange juice

##### 2.2.6.1 Total antioxidant activity (TAA)

The method described by Prieto et al. (1999) was adopted in the determination of total antioxidant activity. The calibration curve solution was prepared by pipetting 0.0, 20.0, 40.0, 60.0, 80.0 and 100.0 μg/ml of ascorbic acid standard solution in triplicates into clean dried test tubes. The samples for the analysis were prepared to a final concentration of 100 μg/ml solution in distilled water. From the standard and samples solution prepared, 100 μl of each was pipetted into separate test tubes and made up to1.0 ml with distilled water to give a 10-fold dilution. One ml of the reagent (consisting of 28 mM trisodiumphosphate and 4 mM ammonium molybdate in 1 l standard flask and made up to mark with 0.6 M sulphuric acid) was added to each standard sample and the blank (prepared by replacing sample with distilled water). The test tubes were capped and incubated at 100 °C for 90 min, cooled to room temperature and absorbance of the reaction mixtures was measured at 695 nm against a reagent blank in the spectrophotometer (INESA, 752N UV-VIS Spectrophotometer). Ascorbic acid calibration curve was obtained by plotting absorbance of the standard solution against concentration and TAA (μgAAE/ml) of the sample was obtained from the curve by extrapolation. The TAA of the sample obtained in μg AAE/ml was expressed in mg AAE/g using Eq. (3);

(3) $$TAA\left(\frac{mgAAE}{g}\right)=\left(\frac{\mu gAAE}{mL}\right)\times \left(\frac{1mg}{1000\mu g}\right)\times \left(\frac{ml\;solvent}{g\;sample}\right)\times dilution\;factor$$

##### 2.2.6.2 DPPH (diphenyl-1-picryhydrazyl) scavenging assay

This was determined using the stable radical DPPH (2, 2-diphenyl-1-picrylhydrazyl hydrate) method as reported by Gulcin et al. (2003). 0.1 mM solution of DPPH in ethanol was prepared and 1 ml of this solution was added 3 ml of the extract solution in water at different concentrations (12.5–62.5 mg/ml). Thirty minutes later, the absorbance was measured at 517 nm. Lower absorbance of the reaction mixture indicates higher free radical scavenging activity. The free radical scavenging ability was calculated using Eq. (4):

(4) $$\;\%\;DPPH=\left(\frac{Abs\;controll-Abs\;sample}{Abs\;control}\right)\times 100\;\left(\%\right)$$

where Abs _control_ is the absorbance of the control reaction (containing all reagents except the test compound), and A_sample_ is the absorbance of the sample extract. Sample concentration was calculated from the graph by plotting inhibition percentage against sample concentration.

##### 2.2.6.3 FRAP assay

This was determined using colorimetric method described by Huyut et al. (2017). Varying concentrations (10, 20, 30 μg/ml) were prepared from the 1 mg/ml stock solutions of the phenolic and flavonoid compounds. The sample volume in the tubes was made up to 0.5 ml with acetate buffer (pH 3.6). Afterwards, 2.25 ml each of FeCl_3_ solution and FRAP reagent was added to each of the tubes to make it up to 5 ml. The tubes were incubated for 10 min and the absorbance of the mixture was read at 593 nm against the blank. Acetate buffer was used as a blank control sample.

##### 2.2.6.4 Metal chelating assay

This was carried out according to the method of Gulcin (2006). Briefly, functional orange juice (5–30 Ag/m) in 0.4 ml was added to a solution of 2 mM FeCl_2_ (0.05 ml). The reaction was initiated by the addition of 5 mM ferrozine (0.2 ml) and total volume was adjusted to 4 ml of ethanol. Then, the mixture was shaken vigorously and left at room temperature for ten minutes. Absorbance of the solution was then measured spectrophotometrically at 562 nm. The percentage inhibition of ferrozine–Fe^+2^ complex formations was calculated using Eq. (5).

(5) $$Metal\;chelating\;activity\;\left(\%\right)=\left(\frac{Abs\;control-Abs\;sample}{Abs\;control}\right)\times 100\left(\%\right)$$

where Abs _control_ = absorbance of control sample (the control contained 1 ml each of FeCl_2_ and ferrozine, complex formation molecules) and Abs _sample_ = absorbance of sample.

#### 2.2.7 Inhibition of linoleic acid oxidation

Linoleic acid oxidation was measured using a slight modification of the method described by Girgih et al. (2011). Samples at final assay concentrations of 0.25, 0.5, and 1.0 mg/ml were dissolved in 1.5 ml of 0.1 M sodium phosphate buffer, pH 7.0. Each mixture was added to 1 ml of 50 mM ethanolic linoleic acid and stored in a glass test tube kept at 60 °C in the dark for 7 days. On a daily basis, 100 μl of the sample mixture was removed and mixed with 4.7 ml of 75% aqueous ethanol, 0.1 ml of ammonium thiocyanate (30%, w/v) and 0.1 ml of 0.02 M acidified ferrous chloride (dissolved in 1 M HCl). An aliquot (200 μl) of the resulting solution was added and the degree of color development was measured using the spectrophotometer at 500 nm after 3 min incubation at room temperature. An increased absorbance implied an increase in the level of linoleic acid oxidation.

### 2.3 Antidiabetic properties of functional orange juice

(i) Inhibition of α-glucosidase activity

The method described by Taslimi and Gulcin (2017) was adopted in the assay of inhibition of α-glucosidase activity. Samples preparation was carried out by dissolving 20 mg in 2 ml (EtOH:H_2_O).). In order to get entire enzyme inhibition, phosphate buffer was used to prepare multiple solutions. Phosphate buffer (75 μl), 20 μl of the enzyme solution in phosphate buffer (0.15 U/ml, pH 7.4) and sample (5 μl). This was followed by initial incubation at 35 °C for 10 min prior to the addition of P-NPG to initiate the reaction. P-NPG (20 μl) was added in phosphate buffer (5 mM, pH 7.4) and then incubated at 35 °C. Acarbose compound was used as positive control. The absorbances were spectrophotometrically measured at 405 nm. One unit of α-glycosidase equals amount of enzyme that catalysed the hydrolysis of 1.0 mol substrate per minute at pH 7.4.

(ii) Inhibition of α-amylase activity

The α-amylase inhibition assay was carried out according to the method of Taslimi and Gulcin (2017). Using Acarbose as the standard inhibitor, 2 g starch was dissolved in 80 mL of NaOH (0.4 M) as the substrate solution, followed by heating for 30 min at 80 °C and subsequent cooling in iced water. The pH of the solution was then adjusted to 6.0 with 2.0 M HCl, then water was added to make up to 100 ml mark. To prepare the sample solution, 20 mg was dissolved in 2 ml (EtOH: H_2_O). Phosphate buffer was used to prepare multiple solutions in order to get entire enzyme inhibition. Substrate (35 μl), phosphate buffer (pH 6.9, 35 μl), and sample (5 μl) solutions were mixed and preincubated at 35 °C for 30 min. Afterwards, 20 μl of a 50 μg/ml enzyme solution was added and the solution incubated for another 30 min. The reaction was terminated by adding 50 μl of 0.1MHCl. The absorbance was measured spectrophotometrically at 580 nm. One unit of α-amylase enzyme equals the amount of enzyme that released 1.0 mg of maltose from starch in 3 min (pH 6.9 at 20 °C).

#### 2.3.1 Storage stability of functional orange juice

The shelf-stability of the functional orange juice samples was evaluated for 90 days**.** During this period, the juice samples were evaluated every 2 weeks for quality characteristics (physicochemical, antioxidant and antidiabetic parameters) using some of the methods described earlier. The functional orange juice samples were stored at ambient temperature under cool and dry environments.

#### 2.3.2 Sensory evaluation of functional orange juice

Sensory characteristics of the fortified juice were evaluated based on colour, taste, flavour, mouthfeel and overall acceptability using a 9-point Hedonic scale. This was done on a scale of 1 (for dislike extremely) to 9 (for like extremely). The taste panel analysis was conducted using 20 semi-trained panelists who are familiar with orange juice. The evaluation was conducted at room temperature in a well-lit and properly ventilated environment. There was water to rinse mouth in between tasting protocols of the samples. The samples were randomly coded and presented as KCF, FKC, KFC and FCK (with isolate/hydrolysate of 1.0%, 0.8%, 0.6% and 0.0% respectively).

##### 2.3.2.1 Confirmation of consent of panelists

The semi-trained panelists selected to participate in the study were made voluntary and confidential. Twenty (20) panelists (comprising of 12 females and 8 males between the age gap of 24–38 years) were selected from University community based on their knowledge about conduct of sensory evaluation exercise and familiarity with the type of product under evaluation.

The panelists gave their consent and confirmed their willingness to participate before taken part in the evaluation exercise.

##### 2.3.2.2 Approval and ethics regulation

The protocol and the instruments (Questionnaire) used were approved by the Department of Food Science and Technology, Obafemi Awolowo University, Ile-Ife Research Committee in line with the approved ethics of the Institutional Research Committee.

#### 2.3.3 Statistical analysis

Experiments were performed in triplicates, and data analyzed were mean subjected to analysis of variance (ANOVA). Means were separated by the Tukey's multiple range test (SPSS version 20) while results were taken to be significant at 5% level (p < 0.05).

## 3 Results and discussion

### 3.1 Physicochemical properties of functional orange juice

Table 1 presents the results of the physicochemical properties of freshly prepared juice fortified with varying proportions of isolate/hydrolysate fractions.

**Table 1:** Physicochemical properties of fortified orange juice samples.

Samples	pH	TTA (%)	Total Sugars (˚Brix)	AA (mgAAE/100 ml)	TPC (mgGAE/100 ml)
IS-A	4.41 ± 0.01^f^	0.59 ± 0.00^c^	9.50 ± 0.00^bcd^	190.48 ± 0.00^a^	46.97 ± 0.00^de^
IS-B	4.45 ± 0.01^c^	0.52 ± 0.00^f^	9.27 ± 0.06^de^	190.48 ± 0.00^a^	35.22 ± 0.00^g^
IS-C	4.48 ± 0.00^b^	0.52 ± 0.00^f^	9.00 ± 0.00^e^	190.48 ± 0.00^a^	29.79 ± 0.00^j^
PE-A	4.24 ± 0.01^l^	0.65 ± 0.00^a^	9.97 ± 0.06^ab^	190.48 ± 0.00^a^	63.70 ± 0.00^a^
PE-B	4.33 ± 0.01^j^	0.60 ± 0.00^bc^	9.77 ± 0.06^abc^	190.48 ± 0.00^a^	52.44 ± 0.00^b^
PE-C	4.35 ± 0.01^i^	0.56 ± 0.00^b^	9.43 ± 0.06^cde^	190.48 ± 0.00^a^	45.92 ± 0.00^e^
PA-A	4.32 ± 0.01^k^	0.60 ± 0.00^b^	10.00 ± 0.00^a^	190.48 ± 0.00^a^	50.90 ± 0.00^c^
PA-B	4.38 ± 0.00^h^	0.55 ± 0.00^e^	9.83 ± 0.06^abc^	190.48 ± 0.00^a^	48.02 ± 0.00^d^
PA-C	4.42 ± 0.00^de^	0.52 ± 0.00^f^	9.63 ± 0.06^abcd^	190.48 ± 0.00^a^	41.66 ± 0.00^f^
TR-A	4.39 ± 0.01^g^	0.53 ± 0.00^g^	9.47 ± 0.06^cde^	190.48 ± 0.00^a^	46.20 ± 0.00^e^
TR-B	4.41 ± 0.01^ef^	0.51 ± 0.00^g^	9.25 ± 0.07^abc^	190.48 ± 0.00^a^	32.91 ± 0.00^h^
TR-C	4.42 ± 0.01^d^	0.50 ± 0.00^g^	9.00 ± 0.00^e^	190.48 ± 0.00^a^	31.57 ± 0.00^i^
CC	4.58 ± 0.00^a^	0.49 ± 0.00^g^	8.50 ± 0.00^f^	190.48 ± 0.00^a^	28.17 ± 0.00^k^

IS: Isolate; PE: Pepsin hydrolysate; PA: Pancreatin hydrolysate; TR: Trypsin hydrolysate; A = 99:1.00 Juice: Isolate/hydrolysate ratio; B = 99.2:0.80 Juice: Isolate/hydrolysate ratio; C = 99.4: 0.60 Juice: Isolate/hydrolysate ratio; CC = 100% Juice.

Values reported are means ± standard deviation of triplicate determinations. Mean values with different superscript within the same column are significantly (p < 0.05) different. TTA: Titrable acidity; AA: Ascorbic acid; TPC: Total phenol contents.

Table 1 showed the orange juice sample pH (a measure the degree of acidity and alkalinity) ranging between 4.24 and 4.58. The orange juice with the highest level of pepsin-hydrolysed protein (PE-A) had the lowest pH (i.e. the highest acidity) while the control sample showed the highest pH (i.e. least acidic). Most of the orange samples exhibited significant difference (p < 0.05) from one another except samples with 0.6% pancreatic hydrolysate as well as those with 0.8% and 0.6% trypsin hydrolysate (PA-C, TR-B and TR-C) which are not significantly different (p > 0.05) from one another. The pH values of the orange juice obtained in this study increased with decreasing levels of fortification. This showed a direct relationship between the levels of isolate/hydrolysate fractions incorporated and the degree of acidity of the orange juice. This study presented comparable range of pH values (4.33–4.58) with the 4.33 recorded by Rekha et al. (2012) but showed lower acidity when compared with 3.50 reported for ripe *Citrus sinensis* juice for (Akusu et al., 2016)*.*

Also presented in Table 1 are the titratable acidity (TTA) levels of the orange juice samples. Titratable acidity is also a measure of acidity in terms of the citric acid equivalence of the orange juice. The TTA values in this study ranged from 0.49% (for the control sample) to 0.65% (for PE-A). In comparison with the control all the samples showed significant difference **(**p < 0.05**)** except those fortified with trypsin-hydrolysed protein (TR-A, TR-B and TR-C). As expected, the TTA values showed a reverse order when compared with the pH values, thus a direct relationship with the proportion of isolate/hydrolysate. This study presented comparable range of values with 0.56% reported by Rekha et al. (2012) for *Citrus reticulata* juice (extracted manually and sieved with muslin cloth) but much lower range compared with 1.01% reported for manually-expressed *Citrus sinensis* nectar (Dhankhar et al., 2019). The observed differences in the pH and TTA values could be attributed to seasonal variation, degree of ripeness as well as the type and extent of nutrients incorporation.

The refractive indices of the juice samples are also indicated in Table 1, this is a measure of the total soluble solids or total sugar (in ºBrix) in the orange juice samples. The values ranged from 8.50 (for the control sample) to 10.00 (for PE-A). All the fortified juice samples were significantly different (p **<** 0.05) from the control sample. A comparable value of 10.0 was reported for manually-extracted sweet orange juice by Akusu et al. (2016) while a much higher value of 14.00 for *Citrus sinensis* nectar was reported by Dhankhar et al. (2019). Although, the control sample in this study exhibited a much lower value as compared with 11.00 reported for India *Citrus sinensis* and 11.78 reported for hand-squeezed Saudi Arabia *Citrus sinensis* (cv. Orlando) by Shravan et al. (2018) and Al-Juhaimi and Ghafoor (2013) respectively. A comparable value of 8.70 was however reported for China *Citrus sinensis* (cv. Range and cv. Jincheng) by Niu et al. (2008). These wide variations in Brix values reported could be as a result of the additives incorporated into the nectar, the orange species, cultivars, regional and environmental conditions, stage of maturity, the extent of ripeness as well as period of harvest.

From Table 1, the ascorbic acid (AA) values of the fresh orange juice samples were not affected by the proportion of isolate/hydrolysate used in this study. The values remained 19.00 mgAAE/100 ml at all the levels of fortification showing that incorporation of protein isolate/hydrolysate into orange juice at these levels has no significant effect on the ascorbic acid content of the fresh orange juice. This value is however higher than the range 36.03–79.19 mgAAE/100 ml reported by Louaileche et al. (2015) for seven citrus juice varieties expressed using a fruit squeezer and centrifuged (for 20 min at 4500rpm and 5 °C).

The total phenol content (TPC) of the orange juice samples is shown in Table 1. The TPC values ranged between 28.17 mgGAE/100 ml (for the control) to 63.70 mgGAE/100 ml (for PE-A) with all the fortified samples being significantly different (p < 0.05) from the control sample. The values obtained in this study were lower range than the 82.00 mgGAE/100 ml and 70.15 mgGAE/100 ml obtained by Rekha et al. (2012) and Dhankhar et al. (2019) for *citrus sinensis* juice.

From the present result, there is an indication that the TPC of the orange juice samples increased with increasing proportions of protein isolate/hydrolysate. This could be as a result of higher proportions of basic and or hydrophobic amino acids with greater tendency to interact with the polyphenol compounds in the orange juice (Zhang et al., 2020). Phenolic compounds can donate hydrogen to the carboxyl group of protein to form hydrogen bond which has the ability to alter their nutritional, physicochemical or even bioactive properties (Ozdal et al., 2013).

### 3.2 Antioxidant and antidiabetic properties of functional orange juice

The antioxidant and antidiabetic properties of freshly-prepared orange juice fortified with varying proportions of isolate/hydrolysate fractions were carried out.

Table 2 shows the Total antioxidant activity (TAA) of orange juice samples. The TAA implies the overall ability (either from phenolic, non-phenolic compounds, ascorbic acids, etc.) to inhibit oxidation. The TAA values ranged from 0.45 to 0.57 with all the samples showing significant difference (p < 0.05) from the control sample. The values showed that the control sample had the lowest TAA value while sample PA-A recorded the highest. This is an indication that addition of the protein isolate/hydrolysate improved the TAA of the orange samples, although with minimal variation with increasing percentage of the isolate/hydrolysate. Moreover, the range of values recorded in this study were comparable to 0.127 mg/ml and 0.48 mg/ml obtained for mechanically-squeezed *Citrus aurantifolia* and persimmon as reported by Shahkoomahally and Ramezanian (2017) and Kumari et al. (2013) respectively. A much higher values of 13.40–14.72 mgAAE/ml were also obtained for manually-extracted cactus pear juice by Mazari et al. (2018). This may be attributed to genetic variation in fruits and the non-specificity of the antioxidant determination method used.

**Table 2:** Antioxidant and antidiabetic properties of fortified orange juice samples.

Samples	TAA (μgAAE/ml)	DPPH (%)	FRAP Fe^2+^ in mMol	M.C (%)	α-AMY (%)	α-GLU (%)
IS-A	0.55 ± 0.00^de^	87.75 ± 0.00^g^	0.53 ± 0.00^e^	45.50 ± 1.10^de^	36.40 ± 0.70^f^	30.85 ± 0.40^g^
IS-B	0.55 ± 0.00^ef^	87.63 ± 0.01^g^	0.52 ± 0.00^f^	44.54 ± 1.10^ef^	34.80 ± 0.30^g^	29.93 ± 0.30^h^
IS-C	0.54 ± 0.00^g^	87.35 ± 0.03^g^	0.51 ± 0.00^f^	43.84 ± 1.10^f^	33.21 ± 0.70^h^	29.18 ± 0.20^i^
PE-A	0.56 ± 0.00^ab^	90.28 ± 0.03^d^	0.61 ± 0.00^b^	48.43 ± 0.80^c^	40.72 ± 0.80^b^	40.23 ± 0.30^b^
PE-B	0.56 ± 0.00^bcd^	89.86 ± 0.03^de^	0.60 ± 0.00^c^	47.15 ± 0.90^c^	39.55 ± 0.40^cd^	38.18 ± 0.50^c^
PE-C	0.55 ± 0.00^f^	89.27 ± 0.04^f^	0.58 ± 0.00^d^	46.84 ± 0.90^cd^	38.54 ± 0.60^de^	36.95 ± 0.30^d^
PA-A	0.57 ± 0.00^a^	90.85 ± 0.00^c^	0.62 ± 0.00^a^	54.85 ± 0.90^a^	42.11 ± 0.30^a^	40.97 ± 0.30^a^
PA-B	0.56 ± 0.00^abc^	89.71 ± 0.03^e^	0.61 ± 0.00^b^	51.43 ± 0.70^b^	40.85 ± 0.60^b^	38.18 ± 0.20^c^
PA-C	0.56 ± 0.00^abc^	89.23 ± 0.03^f^	0.59 ± 0.00^c^	50.08 ± 0.70^b^	40.18 ± 0.80^bc^	37.62 ± 0.30^c^
TR-A	0.56 ± 0.00^abc^	92.68 ± 0.01^a^	0.59 ± 0.00^c^	53.35 ± 1.20^a^	40.30 ± 0.70^bc^	35.47 ± 0.10^e^
TR-B	0.56 ± 0.00^bcd^	92.85 ± 0.04^a^	0.58 ± 0.00^d^	51.64 ± 0.70^b^	39.04 ± 0.60^d^	32.41 ± 0.50^f^
TR-C	0.56 ± 0.00^cd^	92.15 ± 0.03^b^	0.57 ± 0.00^d^	50.18 ± 0.80^b^	37.62 ± 0.70^e^	31.28 ± 0.30^g^
CC	0.45 ± 0.00^h^	69.72 ± 0.02^h^	0.50 ± 0.00^g^	39.19 ± 1.00^g^	28.88 ± 0.60^i^	27.32 ± 0.10^j^

IS: Isolate; PE: Pepsin hydrolysate; PA: Pancreatin hydrolysate; TR: Trypsin hydrolysate; A = 99:1.00 Juice: Isolate/hydrolysate ratio; B = 99.2:0.80 Juice: Isolate/hydrolysate ratio; C = 99.4: 0.60 Juice: Isolate/hydrolysate ratio; CC = 100% Juice.

Values reported are means ± standard deviation of triplicate determinations. Mean values with different superscript within the same column are significantly (p < 0.05) different. TAA: Total antioxidant activity; MC: Metal chelating ability.

Also presented in Table 2 is the DPPH radical scavenging activity (DRSA) of the orange juice samples. The values ranged between 69.72% (for the control sample) to 92.85% (for sample TR-B) with significant difference (p < 0.05) existing between all the samples and the control. The values indicated that trypsin-hydrolysed protein had a more pronounced effect on the DRSA of the orange juice than the pepsin and pancreatin-hydrolysed while the effect was least in the isolate-fortified orange juice sample. The values obtained in this study were higher than 61.35% and 67.78% obtained for hand-squeezed *Citrus sinensis* and mechanically-squeezed *Citrus aurantifolia* by Al-Juhaimi and Ghafoor (2013) and Kumari et al. (2013) respectively. The value recorded for the control sample also reflect the fact that orange juice possesses some inherent free radical scavenging abilities (due to the presence of flavonoids, carotenoids, ascorbic acid, etc.) which were increased by the incorporation of isolate/hydrolysate.

The Ferric-reducing antioxidant power (FRAP) of fortified orange juice samples are presented in Table 2. FRAP is a spectrophotometric assay that determines the ability of a product to reduce ferric ion to its ferrous form through electron donation. The FRAP values ranged between 0.50 to 0.62 mMol with the control sample having the lowest while PA-A recorded the highest value. All the fortified samples were significantly different **(**p **< 0.05**) from the control sample, an indication that fortification improved the FRAP of the orange juice. The FRAP values recorded in this study are comparable with the 0.41mMol for fresh orange juice and 0.15 to 0.46 mMol obtained for *Citrus aurantium* reported by Wern et al. (2016) and Rekha et al. (2012) respectively. The higher FRAP value of the fresh orange juice (CC) compared to those obtained from literature may be due to species and genetic variation of the fruits. Also, the increase in FRAP values with fortification could be attributed to the high reducing power of the sulphur-containing amino acids (methionine and cysteine) as well as the electron-dense nature of the hydrophobic amino acids of the protein to actively donate electrons, thus reducing the ferric cyanide complex (Nwachukwu and Aluko, 2019).

The metal-chelating (MC) ability of fortified orange juice samples is also shown in Table 2. Transition metals such as iron and copper are key promoters of oxidative reactions both in food products and body systems, thus evaluating the ability of natural products to bind them and prevent the resulting deteriorative damages can be used to measure their antioxidant potentials (Girgih et al., 2013). The values recorded in this study ranged from 39.19% for the control sample to 54.85% for PA-A. There was significant difference (p < 0.05) between the control sample and the fortified samples indicating that the incorporation of the protein isolate/hydrolysate as functional ingredient was able to improve the metal-chelating ability of the orange juice. A lower value of 20.9% (at 0.1 mg/ml concentration) was recorded by Divya et al. (2016) for the pulp of *Citrus aurantium* while 7.98 mg EDTA/L of juice was recorded for hand-squeezed, white *Citrus maxima* (Abirami et al., 2014). The difference in values reported could be as a result of different citrus cultivar while the increase in metal-chelating ability with increasing functional ingredients could be traced to the ability of some amino acids (such as the carboxylate groups of aspartic and glutamic acid as well as the ring nitrogen atom of histidine) to bind transition metals.

Also presented in Table 2 is the percentage inhibition of the orange juice samples on the activities of α-amylase and α-glucosidase enzymes. These enzymes catalyse the conversion of starch and sugars in foods to absorbable glucose in the blood stream resulting in an increased blood sugar level which could be detrimental to the health of people suffering from diabetes mellitus. Hence, inhibiting the activities of these enzymes is an important step in the management of this health condition.

From this study, the inhibition of α-amylase and α-glucosidase enzymes ranged between 28.88 and 42.11% and 27.32–40.97% respectively. For the two enzymes, the control samples had the lowest values of inhibition while PA-A recorded the highest values. Also, there were significant differences (p < 0.05) between the control samples and the fortified samples in both cases, an indication that incorporation of protein isolate/hydrolysate had a direct effect on inhibiting the activities of the enzymes. It was noted that the percentage inhibition increased with increasing level of functional ingredients. This agrees with earlier findings that the activities of α-amylase and α-glucosidase enzymes increased with increasing concentration of yellow pea hydrolysate (Awosika and Aluko, 2019) some selected plant extracts (Nair et al., 2013).

The range of values presented in this study is comparable with 36.36% and 27.96% reported for roem seed (*Luffa cylindrical*) alcalase-hydrolysate and tryptic-hydrolysate respectively (Arise et al., 2016). According to Abirami et al. (2014) report, much higher values of 75.55%–79.75% for α-amylase and 70.68% and 72.83% for α-glucosidase activities of *Citrus hystrix and Citrus maxima* respectively were observed. The inhibitory ability on the target enzymes has been linked to the presence of inherent phenolics and flavonoids compounds that act by impairing carbohydrate metabolism and glucose uptake (Abirami et al., 2014; Lin et al., 2016). Also, Olusegun and Emmanuel (2019) reported that cationic or branched chain residue of peptides exert inhibitory effects on α-glucosidase and α-amylase enzymes which play a major role in the digestion of dietary carbohydrates to glucose.

### 3.3 Storage stability of functional orange juice

The effects of storage on functional orange juice samples at ambient temperature under cool and dry environments were evaluated fortnightly for a period of 90 days.

#### 3.3.1 Changes in pH of functional orange juice during storage

**Figure 1:**
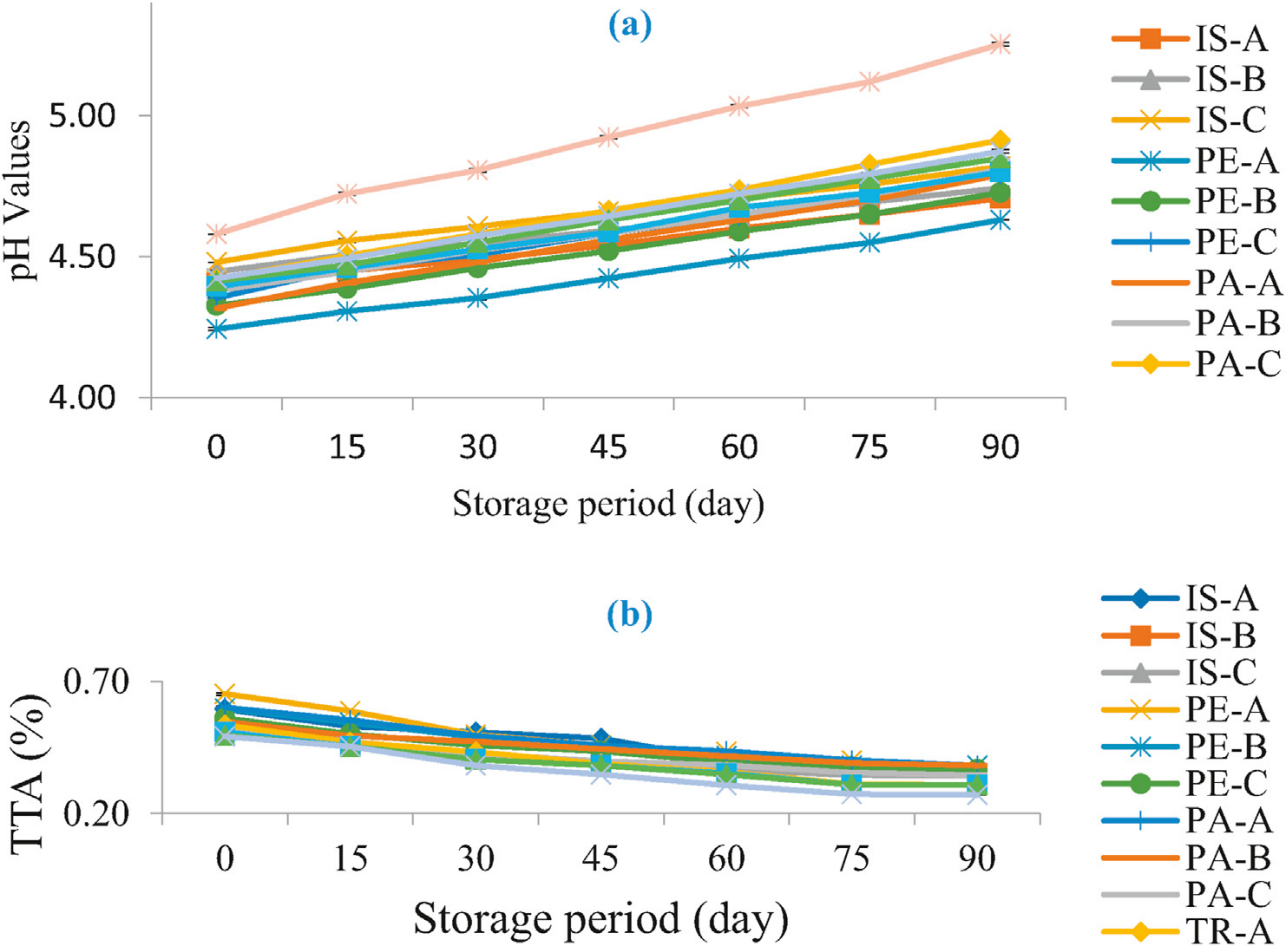
Changes in pH (a) and Titrable Acidity (b) of Functional Orange Juice during Storage. IS: Isolate; PE: Pepsin hydrolysate; PA: Pancreatin hydrolysate; TR: Trypsin hydrolysate; A = 99:1.00 Juice: Isolate/hydrolysate ratio; B = 99.2:0.80 Juice: Isolate/hydrolysate ratio; C = 99.4: 0.60 Juice: Isolate/hydrolysate ratio; CC = 100% Juice.

Figure 1a shows the trend in pH of functional orange juice samples as affected by storage under ambient temperature. pH has long been an important parameter used in quality assessment of food products especially fruit juices. From this study, all the samples including the control showed a steady increase in pH values from storage day 0 to day 90 although the values for the control samples were significantly higher than those of the others throughout the storage period. Sample PE-A showed the lowest values ranging from 4.24 (for day 0) to 4.63 (for day 90) while the control sample showed the highest values ranging from 4.58 (for day 0) to 5.25 (for day 90). The increase in pH values with increasing storage period reported in this study disagrees with Giuffrè et al. (2017) who reported a decrease in pH values of blood orange (3.74–3.34) and concentrated blood orange (3.81–3.50) with increase in storage period. A comparable range of pH value (3.9–5.6) was however recorded for varying blends of soy-enriched orange juice (Kale et al., 2012). The observed increase in pH value with the storage period in this study could be attributed to a decline in the level of acidity due to acid-sugar conversion as the storage period increased.

#### 3.3.2 Changes in titratable acidity of functional orange juice during storage

Figure 1b shows how the titratable acidity of orange juice samples was affected over a 90-day storage period. This usually represents the organic acid component of the juice and in this study, it was measured in terms of the citric acid equivalent of the orange juice. These organic acids are nutritionally excellent and can also find useful application in promoting the shelf life of food products (Singh and Sharma, 2017). As expected, there was an inverse relationship with the pH values obtained i.e. the values for the titratable acidity decreased with the storage period. The resulting values ranged between 0.65% to 0.27%. The control sample had the lowest values (0.49–0.27%) throughout the storage period while the highest values varied between 0.65 to 0.38% among the fortified samples. The values presented in this study compared favourably with the range (0.64–0.064%) reported for soy-enriched orange juice (Kale et al., 2012). The report of Bhardwaj and Nandal (2014) shows a decrease in pH values of Kinnow mandarin juice blends. Gradual oxidation of ascorbic acid and conversion of acid to sugar during storage could be responsible for the decrease in acidity over storage period.

#### 3.3.3 Changes in total soluble solids of functional orange juice during storage

**Figure 2:**
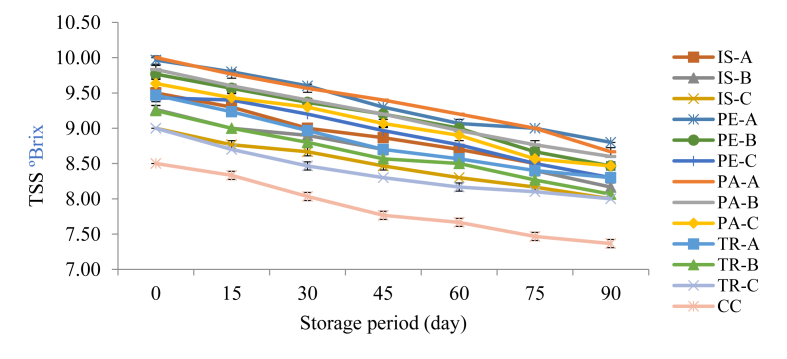
Changes in Total Soluble Solids of Functional Orange Juice during Storage. IS: Isolate; PE: Pepsin hydrolysate; PA: Pancreatin hydrolysate; TR: Trypsin hydrolysate; A = 99:1.00 Juice: Isolate/hydrolysate ratio; B = 99.2:0.80 Juice: Isolate/hydrolysate ratio; C = 99.4: 0.60 Juice: Isolate/hydrolysate ratio; CC = 100% Juice.

The total soluble solids (also TSS or total sugars) of functional orange juice samples is presented in Figure 2. TSS measures the total dissolved solids usually in the form of sugars and acids contained in food products using the mechanism of refractive index of light when passing through different medium. The TSS values recorded in this study reduced consistently with decreasing storage period for all the samples including the control. The control sample had the lowest values (8.50–7.37) which were significantly different (p < 0.05) all through the storage period while PA-A and PE-A exhibited the highest TSS values. These values were slightly lower than the range (10.7–11.5) recorded for blood orange during a 5-month storage period (Giuffrè et al., 2017). Also, decrease in TSS values observed in this study as against earlier reports on blood orange and kinnow mandarin (Giuffrè et al., 2017; Bhardwaj and Nandal, 2014) could be due to the variety as well as stage of maturity and ripeness of the orange fruit.

#### 3.3.4 Changes in ascorbic acid content of functional orange juice during storage

**Figure 3:**
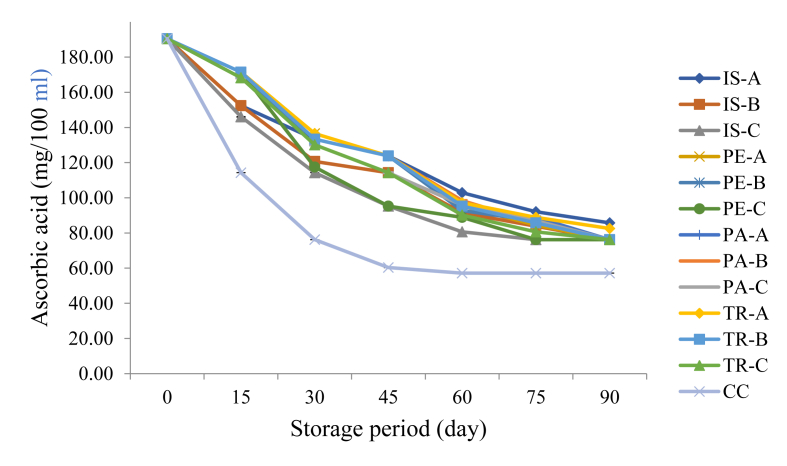
Changes in Ascorbic Acid Content of Functional Orange Juice during Storage. IS: Isolate; PE: Pepsin hydrolysate; PA: Pancreatin hydrolysate; TR: Trypsin hydrolysate; A = 99:1.00 Juice Isolate/hydrolysate ratio; B = 99.2:0.80 Juice: Isolate/hydrolysate ratio; C = 99.4: 0.60 Juice: Isolate/hydrolysate ratio; CC = 100% Juice.

Figure 3 depicts the trend in ascorbic acid (vitamin C) content of fortified orange juice samples during a 90-day storage period. The vitamin C content of fruit juices has been proven to play a vital role in both the nutritional aspect as well as the antioxidant ability of orange juice. Consumption of this vitamin as part of our diet is therefore important as the body system is not capable of synthesizing it. In this study, the vitamin C content of all the samples including the control was 190.48 mg/100 ml at the beginning of the storage period, this shows that incorporation of the functional proteins did not have any effect on the ascorbic acid of the orange juice samples on day zero. However, as the storage period increases, the values for the vitamin C content reduced at varying rates in all the samples. The control samples exhibited the fastest rate (190.48–57.14 mg/100 ml) of decline while IS-A exhibited the slowest rate of decline in vitamin C content.

In other words, all the samples showed a decrease in vitamin C content with storage period with the decrease exhibited by the control sample being significantly lower than that of the fortified samples. Bhardwaj and Nandal (2014) in one of their submissions also reported a decrease in ability of some sulphur-containing amino acids (cysteine and methionine) which reduced the rate of oxidation of ascorbic acid.

#### 3.3.5 Changes in total phenol content of functional orange juice during storage

**Figure 4:**
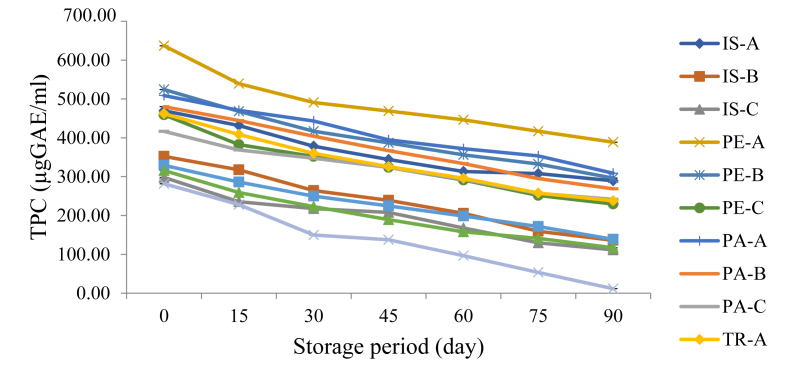
Changes in Total Phenol Content of Functional Orange Juice During Storage. IS: Isolate; PE: Pepsin hydrolysate; PA: Pancreatin hydrolysate; TR: Trypsin hydrolysate; A = 99:1.00 Juice: Isolate/hydrolysate ratio; B = 99.2:0.80 Juice: Isolate/hydrolysate ratio; C = 99.4: 0.60 Juice: Isolate/hydrolysate ratio; CC = 100% Juice.

Figure 4 shows the trend in total phenol content (TPC) of functional orange juice samples over a 90-day storage period. The phenolic compounds mostly found in food products are the flavonoid, anthocyanins, catechins, etc. and are important because they possess antioxidant properties, thus a relationship seems to exist between the TPC and antioxidant properties of food products. In this study, PA-C exhibited the lowest rate in decline (416.64–241.60 μgGAE/m) while the control sample showed the fastest rate of decline (281.74–11.47 μgGAE/m) during the storage period with the decline peak occurring after the 45th day of the storage period.

A similar loss (66–58% at 28 °C and 51–22% at 4 °C) in TPC during storage was also reported by Mgaya-Kilima et al. (2014) for roselle-fruit blends. Giuffrè et al. (2017) however recorded an initial decrease in TPC values from 249.5 mg/L to 230.9 mg/L after 4 months followed by a rise in value to 265.7 mg/L after the fifth month of storage. The decrease in TPC during storage could result from the sensitivity of phenolic compounds to light and oxidation (Ali et al., 2018).

#### 3.3.6 Changes in the total antioxidant activity (TAA) of functional orange juice during storage

**Figure 5:**
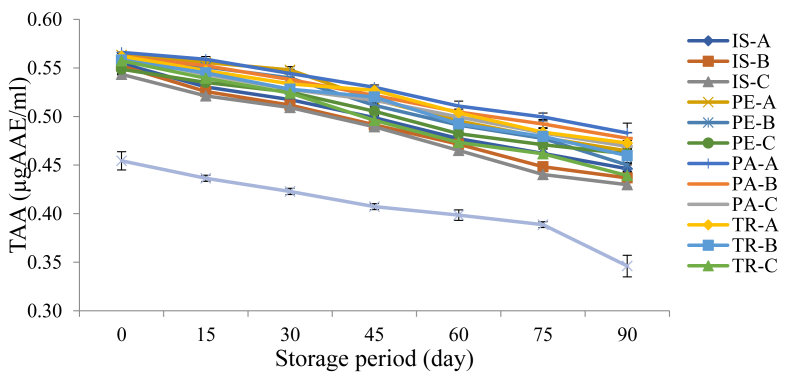
Changes in Total Antioxidant Activity of Functional Orange Juice during Storage. IS: Isolate; PE: Pepsin hydrolysate; PA: Pancreatin hydrolysate; TR: Trypsin hydrolysate; A = 99:1.00 Juice: Isolate/hydrolysate ratio; B = 99.2:0.80 Juice: Isolate/hydrolysate ratio; C = 99.4: 0.60 Juice: Isolate/hydrolysate ratio; CC = 100% Juice.

The pattern exhibited by total antioxidant activity (TAA) of fortified orange juice samples over a 90-day storage period is presented in Figure 5. The TAA measures the overall ability of a host of phytochemical compounds including the phenolics and ascorbic acid to inhibit oxidation. The TAA values in this study decreased with increasing storage period in all the samples however, the control sample showed the lowest values (0.45–0.35 μgAAE/m) while PA-A showed the highest values (0.57–0.48 μgAAE/m) throughout the storage period. The control sample showed a higher rate with values that were significantly different (p < 0.05) all through the storage period, this implies that the functional ingredient was able to reduce the rate at which the samples lost their antioxidant activity. A similar pattern of decreasing TAA was reported for pasteurised orange juice (21%) and pasteurised beet juice (14%) and this was attributed to the reduction in ascorbic acid and phenolic compounds during the storage period (Porto et al., 2017).

#### 3.3.7 Changes in DPPH radical scavenging activity of functional orange juice during storage

**Figure 6:**
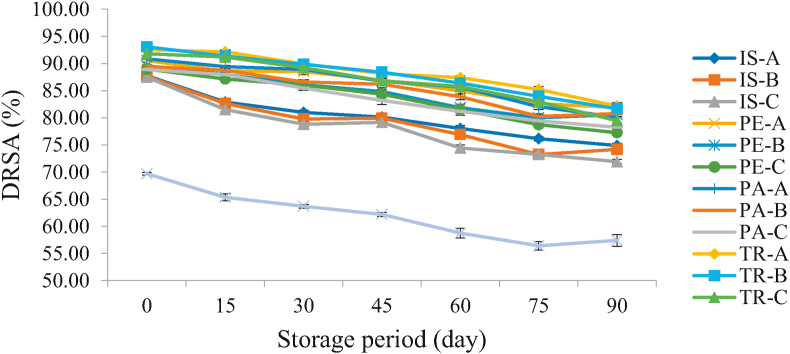
Changes in DPPH Radical Scavenging Activity of Functional Orange Juice during Storage. IS: Isolate; PE: Pepsin hydrolysate; PA: Pancreatin hydrolysate; TR: Trypsin hydrolysate; A = 99:1.00 Juice: Isolate/hydrolysate ratio; B = 99.2:0.80 Juice: Isolate/hydrolysate ratio; C = 99.4: 0.60 Juice: Isolate/hydrolysate ratio; CC = 100% Juice.

Figure 6 shows the trend in the DPPH radical scavenging activity (DRSA) values of fortified orange juice during storage. This study presents a decreasing value of DRSA with increasing storage days however the control samples exhibited the lowest and significantly different (p < 0.05) values all through the storage period. This also implies that the incorporation of the functional proteins into the juice was able to reduce the rate at which the DRSA ability was lost during storage. This can as well be attributed to the relative stability of the added protein isolate and hydrolysate in the orange juice which enhance their earlier-discussed free radical-scavenging ability. A decrease DRSA values during a 5-month storage period was also reported for blood orange juice (from 53.39 to 42.55%) and concentrated blood orange juice (from 39.16 to 33.75%) by Giuffrè et al. (2017). A decrease in DR values of pasteurised orange juice and pasteurised beet juice (by 32% and 37% respectively) during a 30-day storage period was reported by Porto et al. (2017).

#### 3.3.8 Changes in alpha-amylase and alpha-glucosidase enzyme inhibition on functional orange juice during storage

**Figure 7:**
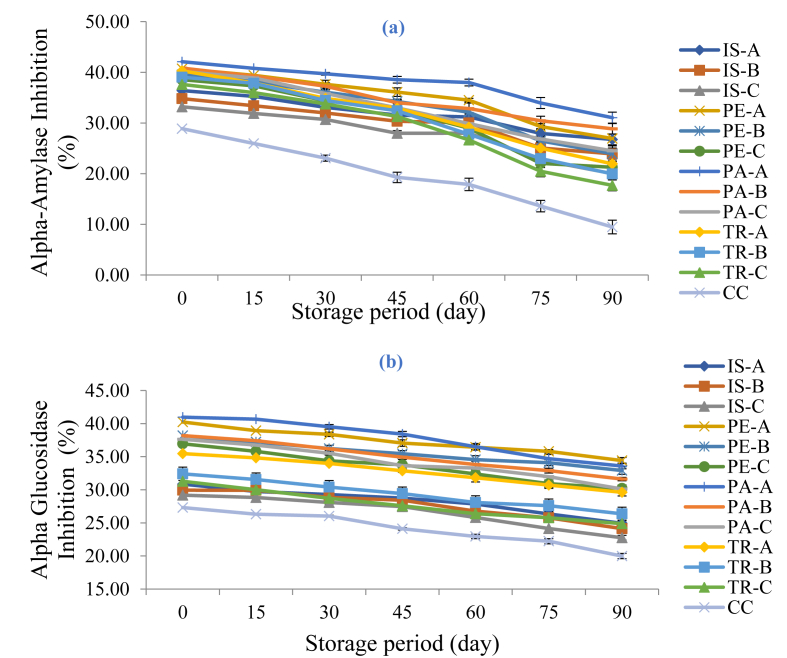
Changes in Alpha-Amylase (a) and Alpha-Glucosidase (b) Inhibition of Functional Orange Juice during Storage. IS: Isolate; PE: Pepsin hydrolysate; PA: Pancreatin hydrolysate; TR: Trypsin hydrolysate; A = 99:1.00 Juice: Isolate/hydrolysate ratio; B = 99.2:0.80 Juice: Isolate/hydrolysate ratio; C = 99.4: 0.60 Juice: Isolate/hydrolysate ratio; CC = 100% Juice.

The *in-vitro* ability of isolate/hydrolysate-fortified orange juice samples to inhibit the activities of alpha-amylase and alpha-glucosidase enzymes over a 90-day storage period are shown in Figure 7 (a and b), respectively. These two enzymes catalyse the conversion of ingested carbohydrate in foods to absorbable glucose in the blood stream. Alpha amylase enzyme is involved in the breakdown of carbohydrates while alpha-glucosidase enzyme breaks down starch and disaccharides to glucose (Nair et al., 2013), thus the complementary actions of these enzymes lead to absorption of glucose into the body. Therefore inhibiting their activities will result in a decrease in the level of glucose in the blood stream, thus providing a way to manage type 2 diabetes (diabetes mellitus) characterised by postprandial rise in blood glucose level. This study showed decreasing activities of both enzymes during the storage period with alpha-amylase enzyme showing a higher rate of loss in activities. The percentage loss of alpha amylase activities ranged between 26.24% (for PA-A) to 67.21% (for the control sample) while that of alpha-glucosidase ranged between 13.80% (for PE-B) to 26.85% (for the control sample). The result showed that the control sample had the highest rate of loss in activities for the two enzymes, an implication that the added functional protein was able to inhibit the enzymes activities more. A higher range of values for both alpha-amylase inhibition (75.55–79.75%) as well as alpha-glucosidase inhibition (70.68–71.88%) was recorded for *citrus spp* (Abirami et al., 2014).

A direct relationship has been reported to exist between alpha-amylase, alpha-glucosidase activities and polyphenol content due to the ability of polyphenols to hydrolyse carbohydrates (Moein et al., 2017), thus the decrease in enzyme activities observed in this study might be due to reduction in phenolic contents as a result of light and temperature during storage (Lin et al., 2016).

### 3.4 Sensory characteristics of isolate/hydrolysate-fortified orange juice

The influence of isolate/hydrolysate fortification on the consumers’ preference for orange juice (in terms of appearance, colour, flavour, mouthfeel and overall acceptability) is presented in Table 3.

**Table 3:** Sensory analyses of Juice:Isolate/Hydrolysate samples.

Functional Ingredient	Code	Appearance	Colour	Flavour	Mouthfeel	Overall acceptability
Isolate	KCF	6.36 ± 0.81^b^	6.55 ± 0.82^a^	6.55 ± 0.82^a^	6.36 ± 0.81^b^	7.18 ± 1.32^a^
	FKC	6.82 ± 0.98^b^	6.91 ± 0.54^ab^	7.73 ± 0.91^a^	7.18 ± 0.60^a^	7.00 ± 0.89^a^
	KFC	8.00 ± 0.89^a^	7.55 ± 0.93^a^	7.09 ± 0.94^a^	7.46 ± 1.04^a^	7.55 ± 0.93^a^
	FCK	7.00 ± 0.78^b^	7.36 ± 1.29^ab^	7.27 ± 1.10^a^	6.91 ± 0.70^ab^	7.27 ± 1.19^a^
Pepsin-HS	KCF	6.91 ± 1.45^ab^	6.55 ± 0.52^b^	5.82 ± 0.75^c^	6.36 ± 0.81^a^	6.55 ± 0.82^a^
	FKC	6.27 ± 0.79^b^	7.27 ± 1.91^ab^	6.46 ± 0.69^b^	6.55 ± 0.82^a^	6.36 ± 0.81^a^
	KFC	7.55 ± 1.13^a^	7.55 ± 0.93^a^	7.46 ± 0.69^a^	6.55 ± 0.93^a^	7.00 ± 0.78^a^
	FCK	7.00 ± 0.63^ab^	6.55 ± 0.52^b^	6.73 ± 0.65^b^	6.50 ± 1.81^a^	6.91 ± 1.58^a^
Pancreatin-HS	KCF	5.63 ± 0.67^c^	5.27 ± 1.01^c^	6.18 ± 1.08^a^	7.18 ± 1.60^a^	6.55 ± 0.52^b^
	FKC	6.64 ± 0.67^b^	6.36 ± 0.81^b^	6.64 ± 0.81^a^	7.09 ± 0.94^a^	7.27 ± 1.19^ab^
	KFC	7.55 ± 0.93^a^	7.46 ± 0.82^a^	7.46 ± 0.82^a^	7.27 ± 1.10^a^	7.55 ± 0.93^a^
	FCK	7.18 ± 1.08^ab^	6.73 ± 0.91^ab^	7.091 ± 1.45^a^	7.182 ± 1.54^a^	6.55 ± 0.52^b^
Typsin-HS	KCF	7.18 ± 1.60^a^	7.18 ± 1.33^a^	6.18 ± 1.40^a^	6.55 ± 1.21^a^	6.36 ± 1.03^a^
	FKC	7.09 ± 0.94^a^	7.00 ± 0.89^a^	6.64 ± 1.03^a^	6.45 ± 1.37^a^	6.72 ± 1.35^a^
	KFC	7.27 ± 1.10^a^	7.27 ± 1.91^a^	6.73 ± 0.91^a^	6.55 ± 0.93^a^	7.00 ± 0.78^a^
	FCK	7.18 ± 1.54^a^	6.73 ± 1.42^a^	6.27 ± 2.00^a^	6.55 ± 1.81^a^	6.91 ± 1.58^a^

KCF = 1.0: 99.0 Juice: Isolate/hydrolysate ratio; FKC = 0.8: 99.2 Juice: Isolate/hydrolysate ratio; KFC = 0.6: 99.4 Juice: Isolate/hydrolysate ratio FCK = 100% Juice. HS: Hydrolysed protein. Values reported are means standard deviation of triplicate determinations. Mean values with different superscript within the same column are significantly (p < 0.05) different.

For the isolate-fortified juice samples, the values for the appearance ranged between 6.36 and 8.00 with KCF recording the lowest and KFC the highest preference. No significant differences (p > 0.05) were exhibited by samples KCF, FKC and FCK (the control) while KFC was significantly different (p < 0.05) from all others. The values for colour (6.55–7.55), flavour (6.55–7.73), mouthfeel (6.36–7.46) and overall acceptability (7.00–7.55) exhibited no significant difference (p > 0.05).

The pepsin-hydrolysate fortified juice samples presented a range of 6.27–7.55 for appearance with only FKC and KFC being significantly different (p < 0.05) from each other. The colour presented a range of 6.55–7.55 with only FKC and KFC being significantly different (p < 0.05) from each other. The values for flavour ranged between 5.82 and 7.46 with samples KCF and KFC being significantly different (p < 0.05) from the control (FCK). The values for mouth feel and overall acceptability showed no significant difference (p > 0.05) among one another and the ranges are 6.36–6.55 and 6.55 to 7.00, respectively.

The values for the pancreatin-hydrolysate fortified juice samples in terms of appearance and colour ranged from 5.63 to 7.55 and 5.27 to 7.46 respectively, with the two sensory parameters showing significant difference (p < 0.05) only between sample KCF and the control (FCK). The respective values for flavour and mouth feel ranged from 6.18 to 7.46 and 7.09 to 7.27 with no significant difference (p > 0.05) among all the samples. Overall acceptability recorded a range of 6.55–7.55 with significant difference existing between only samples KFC and FCK. Trypsin-hydrolysate fortified juice samples indicated no significant difference (p > 0.05) for all the parameters and the range of values recorded were 7.09–7.27 for appearance; 6.73 to 7.27 for colour; 6.18 to 6.77 for flavour; 6.45 to 6.55 for mouth feel and 6.36 to 7.00 for overall acceptability.

Based on the evaluation, most of the judges perceived a very low difference between sample KFC (with the lowest proportion of functional ingredient) and sample FCK (with no added functional ingredient) while samples KCF and FKC recorded higher level of significant difference (p < 0.05) in comparison with the control. KCF similar report was presented for protein-fortified mango juice by Yadav et al. (2016) who explained that sample with highest concentration of hydrolysate had the lowest rating. This may be attributed to the taste imparted by the functional ingredient especially the hydrolysates. As regard the colour and appearance of the juice samples, Bilek and Bayram (2015) also reported that fortifying orange juice with not more than 2.5% hydrolysed collagen gave an acceptable consumer response. It was also observed that samples KCF (with highest concentration of functional ingredients) showed some sediments when left to stand, this was also observed in protein-fortified mango juice as reported by Yadav et al. (2016) who further attributed it to loss of solubility during sedimentation. Although the presence of sediments in sample KCF was not very obvious due to the masking effect by the juice, it could nevertheless be indirectly responsible for the relatively low level of overall acceptability recorded.

The trypsin hydrolysate-fortified samples were more preferred by the panelists in terms of colour and appearance, the pepsin-hydrolysate fortified juice recorded the least preference in terms of flavour, while the isolate hydrolysate-fortified samples were least preferred for mouthfeel. For overall impression, no significant difference (p > 0.05) was recorded except in the pancreatin hydrolysate-fortified samples.

From the responses in this study, it can be deduced that individually, the opinions of the respondents were diverse due to the randomized order of presentation. However, the outcome of the evaluation still pointed to a single direction, an indication that the responses were based wholly on the sensory experience of the panelists.

## 4 Conclusion

It was concluded from this study that production of functional orange juice by incorporating Kersting's groundnut protein isolate and hydrolysate (at the levels of 0.6, 0.8 and 1.0%) was able to improve the overall physicochemical, antioxidant and antidiabetic properties while the sample with the lowest proportion (0.6%) of functional ingredient had the highest sensory acceptability. Also, the shelf stability of orange juice was increased in terms of these properties over a 90-day period. Hence, Kersting's groundnut proteins could find bioactive roles in the production of functional beverages.

## Declarations

### Author contribution statement

Omolayo R. Osungbade: Performed the experiments; Analyzed and interpreted the data; Contributed reagents, materials, analysis tools or data; Wrote the paper.

Abiodun V. Ikujenlola: Conceived and designed the experiments; Analyzed and interpreted the data; Contributed reagents, materials, analysis tools or data; Wrote the paper.

Saka O. Gbadamosi: Conceived and designed the experiments; Analyzed and interpreted the data; Contributed reagents, materials, analysis tools or data.

### Funding statement

This research did not receive any specific grant from funding agencies in the public, commercial, or not-for-profit sectors.

### Data availability statement

Data included in article/supplementary material/referenced in article.

### Declaration of interests statement

The authors declare no conflict of interest.

### Additional information

No additional information is available for this paper.
